# Self-Adaptive Image Reconstruction Inspired by Insect Compound Eye Mechanism

**DOI:** 10.1155/2012/125321

**Published:** 2012-12-30

**Authors:** Jiahua Zhang, Aiye Shi, Xin Wang, Linjie Bian, Fengchen Huang, Lizhong Xu

**Affiliations:** ^1^College of Computer and Information Engineering, Hohai University, Nanjing, Jiangsu 211100, China; ^2^College of Computer Science and Technology, Zhejiang University of Technology, Hangzhou, Zhejiang 310023, China

## Abstract

Inspired by the mechanism of imaging and adaptation to luminosity in insect compound eyes (ICE), we propose an ICE-based adaptive reconstruction method (ARM-ICE), which can adjust the sampling vision field of image according to the environment light intensity. The target scene can be compressive, sampled independently with multichannel through ARM-ICE. Meanwhile, ARM-ICE can regulate the visual field of sampling to control imaging according to the environment light intensity. Based on the compressed sensing joint sparse model (JSM-1), we establish an information processing system of ARM-ICE. The simulation of a four-channel ARM-ICE system shows that the new method improves the peak signal-to-noise ratio (PSNR) and resolution of the reconstructed target scene under two different cases of light intensity. Furthermore, there is no distinct block effect in the result, and the edge of the reconstructed image is smoother than that obtained by the other two reconstruction methods in this work.

## 1. Introduction

The classical reconstruction methods include the nearest neighbor algorithm, bilinear interpolation, and bicubic interpolation algorithm [1, 2]. According to existing research, the reconstruction accuracy of bilinear interpolation is higher than that of the nearest neighbor algorithm, and the former can get better image reconstruction results. However, the reconstructed image by bilinear interpolation appears saw-tooth and blurring sometimes [3]. Although the reconstruction results of bicubic interpolation are better than the others, they always lose efficiency and take much more time. As a compromise, bilinear interpolation is often used for research. These algorithms can improve the reconstruction quality of the original image to some extent. However, only the correlation between the local and global pixels is considered in these algorithms. Interpolation-based reconstruction methods do improve the effect of image reconstruction, but they destroy the high-frequency detailed information of the original image [4, 5]. 

**Figure 1:**
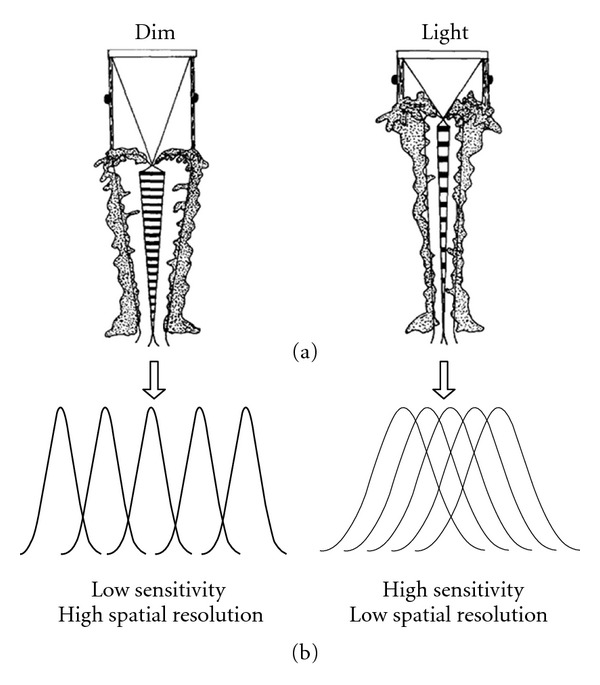
Light-dim adaptive regulatory mechanism of ommatidia. (a) Structure adaptation in ommatidia visual system. (b) Adaptation in the view-field of ommatidia and compound eyes.

Some studies have found that insects have a relatively broad living environment, for instance, the mantis shrimp can live between 50 m and 100 m depth underwater. In such living environment, the light condition changes dramatically, due to the combined effect of sunlight and water media. To adapt to the changing environment, this species, whose ommatidia structure is fixed, must regulate the light acceptance angle adaptively [6, 7]. Through the joint action of the lens and the rhabdome, the mantis shrimp has different degrees of overlapping images in the whole region of the ommatidia. The ommatidia get the different optical information depending on the different lighting conditions. Under the light and the dim environment conditions, the mantis shrimp can regulate the length of rhabdome and lens through relaxing or contracting the myofilament. Based on the biological mechanism above, the ommatidia visual field can be narrowed or expanded to get a relatively stable number of incoming photons and a better spatial resolution. Ultimately, the imaging system can reach balance between the visual field and the resolution [8], as shown in Figure 1. According to Schiff's [9] research, the imaging angle and visual field of the mantis shrimp ommatidia both change while the light intensity condition changes. For instance, the ommatidia visual field is 5° under dim-adapted pattern, but the corresponding visual field will be only 2° under bright-adapted pattern, and some other species also have similar characteristics [10–14]. 

Recently, the compressed sensing theory provides a new approach for computer vision [15–17], image acquisition [18, 19], and reconstruction [20–22]. This method can get the reconstruction results as effectively as the traditional imaging systems do, or even higher quality (in resolution, SNR, etc.), with fewer sensors, lower sampling rate, less data volume, and lower power consumption [23–27]. According to the compressed sensing theory, the compressive sampling can be executed effectively if there is a corresponding sparse representation space. Currently, the compressed sensing theory and application of the independent-channel signal have been developed in-depth, such as single-pixel camera imaging [28]. 

By the combined insect compound eye imaging mechanism with compressed sensing joint sparse model (JSM-1) model [29–32], we use the spatial correlation of multiple sampled signals to get the compressive sampling and reconstruction. Inspired by the light-dim self-adaptive regulatory mechanism of insect compound eyes (ICE), this paper proposes an ICE-based adaptive reconstruction method (ARM-ICE). The new method can execute multiple compressive sampling on the target scene. According to the environment light intensity, it can regulate the sampling visual field to control imaging. The simulation results show that, in contrast to the image-by-image reconstruction and bilinear interpolation algorithm, the new method can reconstruct the target scene image under two kinds of light intensity conditions with higher-peak signal-to-noise ratio (PSNR). The new method also improves the resolution and detailed information of reconstruction. 

In the first section, we describe the imaging control mechanism of insect compound eyes, compressed sensing theory, and current research of bionic compound eyes imaging system.  Section 2 demonstrates the ARM-ICE imaging system pattern from three aspects: visual field self-adaptive adjusting, sampling, and reconstruction.  Section 3 completes the ARM-ICE system simulation under the dim and light conditions and then analyzes the imaging results and the comparison of relevant parameters. In Section 4, we conclude with possible topics for future work. 

## 2. Compressed Sensing-Based Arm-Ice Imaging System Pattern

**Figure 2:**
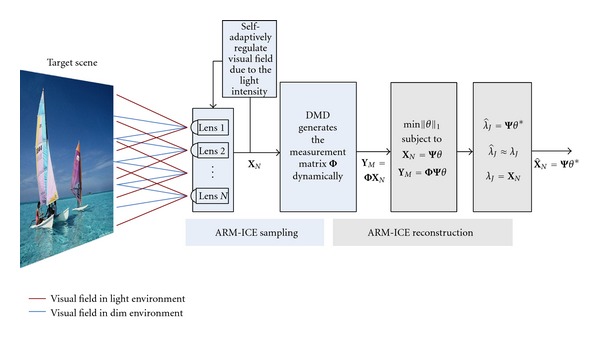
ARM-ICE imaging system pattern.

Figure 2 shows an ARM-ICE imaging system pattern. The purple lines represent the light environment visual field, while the blue lines represent the dim environment visual field. The target scene is imaged, respectively, by the compound eye lens array. The isolation layer is composed by multichannel opening shade blocks, which can be controlled. And each port of shade blocks is connected to a corresponding little lens of compound eye lenses. This structure sets a number of independent controllable light-sensitive cells. Each port of isolation layer opens at different time. The feedback signal controls them to regulate the relative position to make the light from target scene to the n light-sensitive cells. The corresponding area is sparsely sampled in the digital micromirror device. Measurement data can be obtained in the imaging plane. Ultimately, the processor reconstructs the target scene according to the *k*-sparse property of data sensed on the wavelet basis Ψ and the uncorrelated measurement matrix Φ.

### 2.1. Arm-ICE Visual Field Self-Adaptive Regulation

According to the biological research, in the insect compound eyes system under different light intensities, the angle of imaging and the visual field change accordingly [33–37]. Inspired by this self-adaptive ability, this paper mimics the insect compound eye system on its imaging control mechanism based on light intensity sensitivity, to expand or narrow the scope of visual field and overlapping field by regulating the position of the lenses. 

According to the results of biological research, the relationship between light intensity, imaging pore size, and other factors can be described as (1), hereby to regulate the lenses position to achieve the overlap visual field [12]

(1) $$\begin{array}{c} \Delta \rho_{T}=\frac{0.530}{\upsilon_{\max}}\sqrt{\ln cN_{p}-\frac{1}{2}\ln\left[N_{p}+\sigma_{D}^{2}\right],} \end{array}$$

where Δ*ρ*_*T*_ indicates the visual field range, *υ*_max⁡_ indicates the maximum detectable spatial frequency, which can be regarded as a constant, *c* is the mean contrast of the scene, *N*_*p*_ indicates the number of the photons captured by an input port, and *σ*_*D*_^2^ shows the total variance for environmental light intensity. 

From (1), the visual field can be calculated according to the *υ*_max⁡_ set while the light intensity changes. Based on the biological principle above, the visual field range can be regulated according to the environment light intensity.

### 2.2. Compressive Sampling

The digital micromirror device (DMD) senses the optical information from the lenses array, and then makes sparse sampling. The principle is inner product the optical signal from the lenses array perception **X**(*m*) and DMD measurement basis vector **φ**(*m*), and make the result as the output voltage (*v*)*m* of the DMD device at the moment *m*. The output voltage *v*(*m*) of the photodiode can be expressed as the inner product of the desired image *x* with a measurement basis vector [26, 28, 29]:

(2) $$\begin{array}{c} v\left(m\right)\propto \langle X\left(m\right),\varphi \left(m\right)\rangle +O_{\text{DC}}, \end{array}$$

where the value of **φ**(*m*) is related to the position of DMD micro-mirror; when the micromirror turns +10°, *ϕ*_*i*_(*m*) = 1; when the micromirror turns −10°, *ϕ*_*i*_(*m*) = 0. *O*_DC_ is the direct current offset, which can be measured by setting all mirrors to −10°.

Based on the principle of measurement matrix of a single DMD device, we can use the DMD device array to get sparse signals of image system. The compound eye lenses and the isolation layer constitute *n* light-sensitive independent cells, each of which is controlled by the isolation layer to open at different time. The array jointly senses the target scene data *X*_*i*_:

(3) $$\begin{array}{c} X_{i}=X_{i,C}+X_{i,S}, \end{array}$$

where *X*_*i*,*C*_ expresses the common information of the perception data and *X*_*i*,*S*_ expresses the specific information of each lens. Vector **X**_*N*_ = (*X*_1_, *X*_2_,…, *X*_*N*_)^*T*^ indicates the perception data from *n* light-sensitive units. The perception data can be regarded as *k*-sparse on wavelets basis Ψ due to the spatial correlation:

(4) $$\begin{array}{c} X_{N}=\Psi \theta , \end{array}$$

where ***θ*** = (*λ*_0_,*γ*_0_,*γ*_1_,…,*γ*_*J*−1_)^*T*^ is the sparse vector coefficient, consisting of the high-frequency subset *γ*_0_, *γ*_1_,…, *γ*_*J*−1_ (*γ*_*k*_ is subset at scale *J* − *k*) and the low-frequency subset *λ*_0_ of wavelet transform. After light-sensitive lenses obtain *X*_*N*_, *k*-sparse signal *X*_*N*_ is used to generate *M* measurement data of the image plane from the *M* × *N* measurement matrix Φ on the DMD device:

(5) $$\begin{array}{c} Y_{M}={\left(Y_{1},Y_{2},\ldots ,Y_{M}\right)}^{T}=\Phi X_{N}, \end{array}$$

where matrix Φ is a 0-1 matrix, which consists of the output voltage *v*(*m*) of the DMD device in (2) at the moment *m*. Equation (5) can also be described as follows:

(6) $$\begin{array}{c} \left[\begin{array}{c} Y_{1}\\ Y_{2}\\ \vdots \\ Y_{M} \end{array}\right]=\left[\begin{array}{cccc} \Phi_{1} & & & 0\\ & \Phi_{2} & & \\ & & \ddots & \\ 0 & & & \Phi_{M} \end{array}\right]\left[\begin{array}{c} X_{1}\\ X_{2}\\ \vdots \\ X_{N} \end{array}\right]. \end{array}$$

### 2.3. Joint Reconstrucion

According to the multichannel captured data, which are *k*-sparse on wavelet basis and the inconsistency of the measurement matrix Φ with the wavelet basis Ψ, the processor runs the decoding algorithm to reconstruct the target scene:

(7) $$\begin{array}{c} \min{||\theta ||}_{0},\;\text{subject to}{\;Y}_{M}=\Phi \Psi \theta . \end{array}$$

The optimized sparse solution θ* can be gotten by solving the issue of optimizing *l*_0_ norm. The reconstruction of captured data from each lens can be indicated as follows: ${\hat{X}}_{N}={\left({\hat{X}}_{1},{\hat{X}}_{2},\ldots ,{\hat{X}}_{N}\right)}^{T}=\Psi \theta^{*}$. An important issue during the reconstruction process is how to calculate the wavelet basis Ψ. Assume the set of captured data *X*_*N*_ is already known, and *λ*_*J*_ = *X*_*N*_. Each light-sensitive sensor captures the target scene from different views, so its obtained data can be divided into two parts: the common part *λ*_*J*,*P*_ and the particular part *λ*_*J*,*D*_. *T* indicates the lifting wavelet transform after *J* times' recursion:

(8) $$\begin{array}{c} \text{for}\;k=J\;\text{to}\;1\{\begin{array}{c} \lambda_{k-1}=\lambda_{k,P}+U\left(\gamma_{k-1}\right),\\ \gamma_{k-1}=\lambda_{k,D}-P\left(\lambda_{k,P}\right),\\ T\left(\lambda_{k}\right)=\left(\lambda_{k-1},\gamma_{k-1}\right), \end{array} \end{array}$$

where *λ*_*k*−1_ is the low-frequency coefficient set, *γ*_*k*−1_ is the high-frequency coefficient set, *P* is the linear prediction operator, and *U* is the linear update operator. Using the spatial correlation of captured data, *λ*_*k*,  *D*_ can be calculated by *λ*_*k*,*P*_. *γ*_*k*−1_ contains fewer information relatively.

For *λ*_*k*_, after *k* times' recursive lifting wavelet transform:

(9) $$\begin{array}{c} T^{k}\left(\lambda_{k}\right)=\left\{\lambda_{0},{\hat{\gamma }}_{0},{\hat{\gamma }}_{1},\ldots ,{\hat{\gamma }}_{k-1}\right\}. \end{array}$$

After resetting the wavelet coefficients which are under threshold value in *γ*_*i*_, the sparsely structured ${\hat{\gamma }}_{i}$ can be used to reconstruct the original signal *λ*_*k*_ exactly. Assuming that *T* ^−*k*^(•) is a lifting wavelet inverse transform, as the linear prediction operator and the linear update operator are both linear operations, therefore*T* ^*k*^(•) and *T* ^−*k*^(•) are both linear transforms. *T* ^−*k*^(•) can be expressed as follows:

(10) $$\begin{array}{c} T^{-K}\left(\lambda_{0},{\hat{\gamma }}_{0},{\hat{\gamma }}_{1},\ldots ,{\hat{\gamma }}_{k-1}\right)={\hat{\lambda }}_{k},\\ {\hat{\lambda }}_{K}=\Psi \theta^{*}\approx \lambda_{k}, \end{array}$$

where $\theta^{*}={\left(\lambda_{0},{\hat{\gamma }}_{0},{\hat{\gamma }}_{1},\ldots ,{\hat{\gamma }}_{k-1}\right)}^{T}$. Since *λ*_*J*_ = **X**_*N*_, the initial data ${\hat{X}}_{N}=\Psi \theta^{*}$ can be reconstructed exactly.

## 3. Four-Channel Arm-ICE Imaging System Pattern Simulation

**Figure 3:**
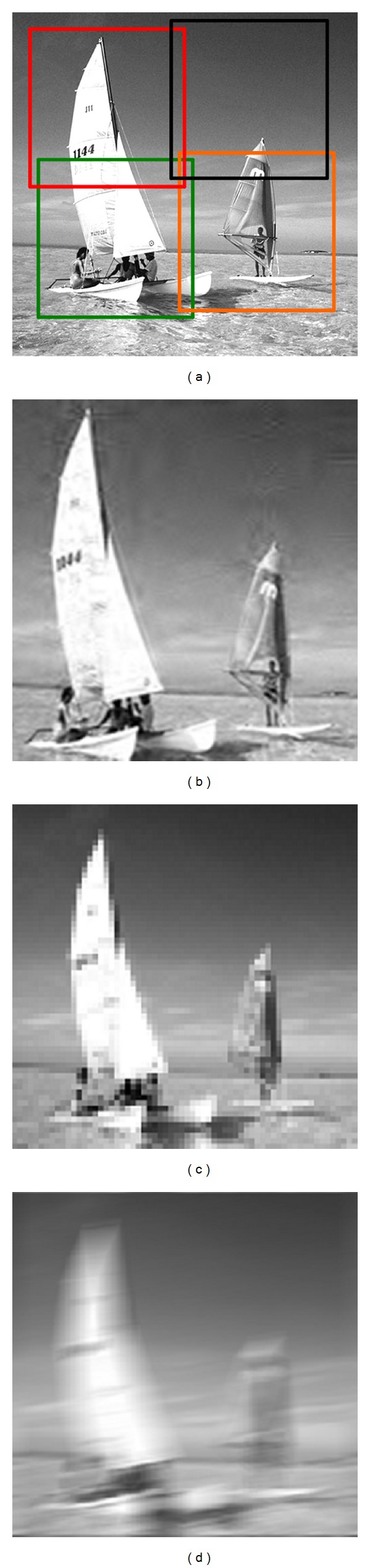
ARM-ICE imaging results and comparison under strong light: (a) target scene, whose brightness value is 144.8527 Nits; (b) ARM-ICE reconstructed image, whose PSNR is 41.9113 dB; (c) result of bilinear interpolation reconstruction, whose PSNR is 34.9112 dB; (d) result of image-by-image reconstruction, whose PSNR is 27.8246 dB.

According to the ARM-ICE visual field self-adaptive adjustment mechanism under different surrounding light intensities described in Section 2.1, in this section, we simulate a four-channel ARM-ICE imaging system. When the surrounding light intensity turns strong, the lenses array regulates their relative positions according to (1) automatically. The simulation results are shown in Figure 3; Figure 3(a) is the target scene under strong illumination environment, whose brightness value is 144.8527 Nits.  Figure 3(b) is the joint reconstruction image from photoelectric coupler array, and its reconstructed PSNR is 41.9113 dB.  Figure 3(c) is a reconstructed image by linear interpolation method, and its PSNR is 27.8246 dB under the same sampling rate as ARM-ICE.  Figure 3(d) is an image-by-image reconstruction, and its PSNR is 27.8246 dB under the same sampling rate as ARM-ICE. 

**Figure 4:**
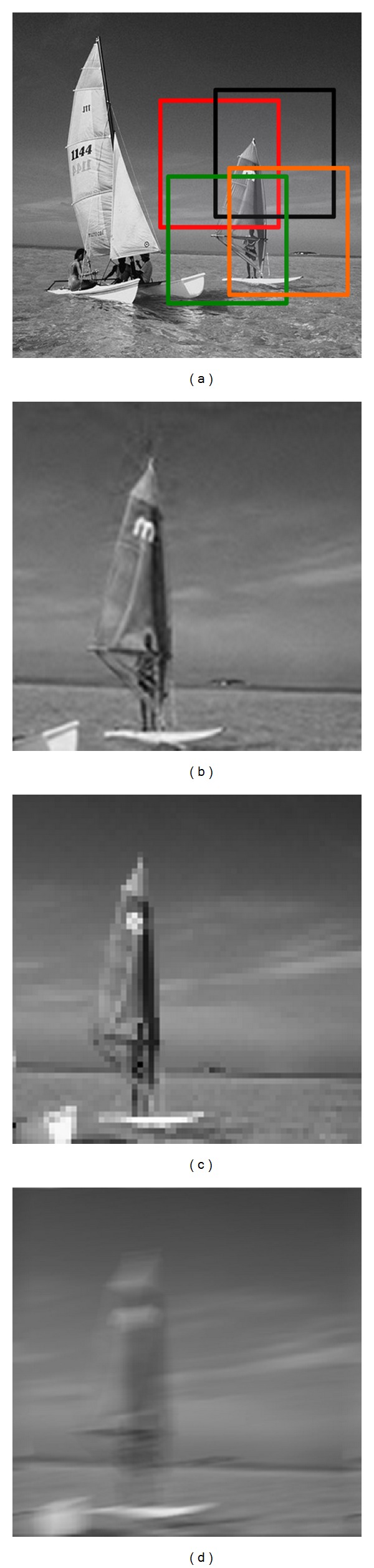
ARM-ICE imaging results and comparison under low light: (a) target scene, whose brightness value is 103.3661 Nits; (b) ARM-ICE reconstructed image, whose PSNR is 44.4705 dB; (c) result of bilinear interpolation reconstruction, whose PSNR is 36.5021 dB; (d) result of image-by-image reconstruction, whose PSNR is 29.5852 dB.

When the surroundings are dim, the compound eye lenses array contracts to the central area, sacrificing the visual field to improve the reconstruction resolution of target scene. The simulation results are shown in Figure 4.  Figure 4(a) is the target scene under the dim conditions whose brightness value is 103.3661 Nits. Put the brightness values into (1) and calculate the lenses' positions at the moment.  Figure 4(b) is the joint reconstruction image from photoelectric coupler array, and its reconstructed PSNR is 44.4705 dB.  Figure 4(c) is the reconstructed image by linear interpolation method. PSNR is 36.5021 dB at the same sampling rate.  Figure 4(d) is the reconstruction result of image-by-image, whose PSNR is 29.5852 dB. 

From the reconstruction effect, the result of linear interpolation method is superior to the result reconstructed by image-by-image. However, there is still obvious block effect, and lack of smoothness at the edge direction. Correspondingly, the image reconstructed by ARM-ICE has a significant improvement in resolution. From Figures 3 and 4, we can see that there is no distinct block effect in the result and the edges of the reconstructed image are smoother compared to the results of the other two reconstruction methods studied in this work. 

**Figure 5:**
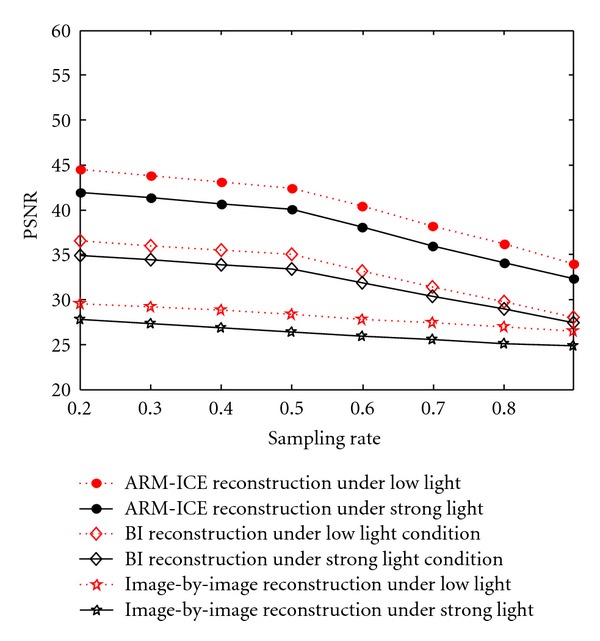
The comparison of PSNR-Sampling rates under low light and strong light conditions.

Figure 5 is the comparison of PSNR-Sampling rates under low light and strong light conditions (144.8527 Nits). The three black lines in the figure show the comparison results under the strong light condition, in which the black dotted line shows the result of ARM-ICE, the black diamond line shows the result of bilinear interpolation, and the black five-pointed star-shaped line shows the result of image-by-image reconstruction. It can be concluded from the figure that the PSNR of ARM-ICE is higher than bilinear interpolation and image-by-image reconstruction under different sampling rates under the strong light condition. 

The three red lines in the figure show the comparison obtained under the low light condition (103.3661 Nits), in which the red dotted line shows the result of ARM-ICE reconstruction, the red diamond line shows the result of bilinear interpolation, and the red five-pointed star-shaped line shows the result of image-by-image reconstruction. It can be seen from the figure that when the target scene is under low light condition, the PSNR of ARM-ICE at different sampling rates is higher than bilinear interpolation and image-by-image reconstruction.

## 4. Conclusion 

Inspired by the imaging mechanism and the adaptive regulatory regulation mechanism of the insect compound eyes, this paper proposes a reconstruction method, which regulates the scale of the sampling area adaptively according to the surrounding light intensity condition. The imaging system pattern of the new method can complete the multichannel independent sampling in the target scene almost at the same time. Meanwhile, the scale of the sampling area and the optical signal redundancy can be regulated adaptively to achieve the imaging control. Compared with the traditional methods, the resolution of the reconstructed image by ARM-ICE method has been significantly improved. The reconstructed image with the proposed method has three features: higher resolution, no distinct block effect, and smooth edge. 

Simulation results indicate that the new method makes the PSNR of the reconstructed image higher under two kinds of light conditions. However, the reconstruction quality under low light conditions is improved by the proposed algorithm at the cost of the scale of the visual field. Therefore, the key issue in the future work would be how to reconstruct high-resolution large scenes in low light conditions.
